# Post-mortem findings in critically ill patients treated with continuous renal replacement therapy

**DOI:** 10.1186/2197-425X-3-S1-A841

**Published:** 2015-10-01

**Authors:** IA Crippa, J Gleeson, V Fontana, J-L Vincent, J Creteur, FS Taccone

**Affiliations:** Erasme University Hospital, Bruxelles, Belgium

## Introduction

Despite the high incidence of acute kidney injury (AKI) in critically ill patients, studies evaluating histopathological renal findings in these patients yielded inconsistent results. No studied specifically evaluated renal histology in intensive care unit (ICU) patients treated by continuos renal replacement therapy for severe AKI.

## Objectives

To describe histopathological findings in patients treated with CRRT for severe AKI.

## Methods

We retrospectively identified all patients admitted to our department of Intensive Care during a two-year period (2012-2013) who required CRRT at any time during ICU stay, who died and had *post-mortem* (PM) examination. Predefined exclusion criteria were: history of end-stage kidney disease requiring dialysis before admission, CRRT required for any reason other than kidney failure and death in the first 24 hours of admission. Kidney histology examination was by light microscopy only.

## Results

Thirty-two post-mortem examinations were performed within 24 hours of death. Mean age was 63 [53-67] years; 12/32 patients (37%) had arterial hypertension and 9/32 patients (28%) had past medical history of chronic kidney disease. The most common ICU diagnosis was severe sepsis/septic shock (14/32; 44%), cardiogenic shock (6/32; 19%) or liver failure (3/32; 9%). *Post-mortem* autolysis precluded analysis in 4 patients and these were excluded from further analysis. Acute tubular necrosis (ATN) was the clinical diagnosis in 21/28 (75%) patients. However, histological features of ATN were only seen in 5 of these. There was significant discordance between the clinical and histological renal diagnosis in 6 patients; 3 had unexpected chronic pyelonephritis, 1 had bilateral renal infarction, 1 had septic emboli and 1 had acute interstitial nephritis. Background atherosclerotic reno-vascular disease was common (32%). Nine patients had normal kidneys at PM examination (32%).

## Conclusions

ATN is a rare pathological finding in renal PM analysis of ICU patients treated with CRRT for severe AKI. One fifth of patients had an unanticipated renal pathology. Normal renal parenchyma was found in one third of all patients.

